# Integrating Iso-seq and RNA-seq data for the reannotation of the greater amberjack genome

**DOI:** 10.1038/s41597-024-03495-7

**Published:** 2024-06-22

**Authors:** Yuanli Zhao, Zonggui Chen, Meidi Hu, Hairong Liu, Haiping Zhao, Yang Huang, Mouyan Jiang, Shengkang Li, Guangli Li, Chunhua Zhu, Wei Hu, Daji Luo

**Affiliations:** 1 Key Laboratory of Breeding Biotechnology and Sustainable Aquaculture, Institute of Hydrobiology, The Innovative Academy of Seed Design, Hubei Hongshan Laboratory, Guangdong Laboratory for Lingnan Modern Agriculture, Chinese Academy of Sciences, Wuhan, 430072 China; 2 Academy for Advanced Interdisciplinary Studies, Peking University, Beijing, 100871 China; 3 Fisheries College, Ocean University of China, Qingdao, 266001 China; 4 China Southern Marine Science and Engineering Guangdong Laboratory (Zhanjiang), Zhanjiang, 524025 China; 5 Fisheries College of Guangdong Ocean University, Guangdong Research Center on Reproductive Control and Breeding Technology of Indigenous Valuable Fish Species, Guangdong Provincial Key Laboratory of Aquatic Animal Disease Control and Healthy Culture, Zhanjiang, 524088 China; 6 Guangdong Provincial Key Laboratory of Marine Biology, Shantou University, Shantou, 515063 China

**Keywords:** Agricultural genetics, Gene expression, Genome informatics

## Abstract

The greater amberjack is a very important fishery species with high commercial value, and it is distributed worldwide. Transcriptome-based studies on *S. dumerili* have been limited by an inadequate reference genome and a lack of well-annotated full-length transcripts. In this study, a total of 12 tissues from juvenile and adult fish both sexes were collected for next-generation RNA sequencing (RNA-seq) and full-length isoform sequencing (Iso-seq). For Iso-seq, a total of 163,218, 149,716, and 189,169 high-quality unique transcript sequences were obtained, with an N50 of 5,441, 5,255, and 5,939, from juvenile, adult male and adult female *S. dumerili*, respectively. We integrated the Iso-seq and RNA-seq data to construct a comprehensive gene annotation and systematically profiled the dynamics of gene expression across the 12 tissues. Our gene models had greater detail and accuracy than those from NCBI and Ensembl, with more precise polyA locations. These resources serve as a foundation for functional genomic studies and provide valuable insights into the molecular mechanisms underlying the development, reproduction and commercial traits of amberjack.

## Background & Summary

The greater amberjack (*Seriola dumerili*), which is known for its rapid growth and superior flesh quality, is an important aquatic species with high commercial value, and it is widely distributed around the world^1^. Furthermore, *S. dumerili* is known to have higher contents of health-promoting fatty acids, such as docosahexaenoic acid (DHA), eicosapentaenoic acid (EPA), and ω-3 polyunsaturated fatty acids (ω-3 PUFAs), than other fish species, making it a preferable choice for a health-conscious diet^2^. Recently, the genomes of *S. dumerili*^3^ and three others different *Seriola* species, Japanese amberjack (*S. quinqueradiata*)^4^, Almaco jack (*S. rivoliana*)^5^ and yellowtail amberjack (*S. lalandi*)^6^, have been successfully sequenced and assembled. Deep sequencing has revolutionized the fields of biology and aquaculture, offering isoform precision and high-throughput capabilities for understanding candidate genes that control commercial traits^7^. However, the greater amberjack genome has currently been elucidated only at the scaffold level^3^, and the available gene models in this genome are mostly derived from computational prediction, which can be incomplete or even inaccurate. This incomplete gene annotation and transcriptome information limits the investigations of the molecular mechanisms involved in various biological processes in the greater amberjack.

Current gene annotations for the greater amberjack, found in database such as NCBI and Ensembl, primarily rely on *in silico* prediction and were assembled using a limited number of short-read RNA-sequencing (RNA-Seq) data^3,8^. These technologies, limited by short read fragment lengths, traditionally fail to capture the full contiguous sequence of RNAs, leading to incomplete, or incorrectly compressed isoform annotations^9^. The recent advancement of long-read isoform sequencing (Iso-seq) can accurately capture full-length (FL) isoforms, utilizing long reads that are greater than 10 kb in length. Iso-seq has been successfully used to discover diverse novel isoforms and annotates full-length isoforms from start to end in a wide variety of species^10^. Many studies have combined Iso-seq and short-read RNA-seq to enhance the accuracy and obtain a more comprehensive understanding of diverse isoform expression quantification^11,12^. Thus, there is a need for an integrated application using Iso-seq and RNA-seq to explore the complexity of transcriptomes in the greater amberjack.

By integrating FL isoform structures revealed by Iso-seq with the deep sequencing coverage of RNA-Seq, we constructed a more comprehensive view of isoform information for 12 tissues representing juvenile and adult stages of both sexes in the greater amberjack. These tissues included the eye, brain, pituitary, heart, muscle, spleen, gill, liver, intestine, stomach, gonad, and kidney. Our findings uncovered 105,607 novel spliced isoforms of known genes and new isoforms transcribed from 13,827 novel gene loci; these discoveries indicate that the amberjack transcriptome is significantly more intricate and dynamic than current annotations released. These data and findings provide a valuable resource for further exploration of critical genes and the molecular mechanisms underlying biological processes and commercial traits of amberjack.

### Ethics statement

All the experiments were conducted according to the guidelines and regulations outlined by the ‘Management and Use of Laboratory Animals of Hubei Province’ and complied with China’s existing laws and regulations for biological research. This study did not involve any endangered or protected species.

## Methods

### Sample collection, library construction and sequencing

**Fig. 1:**
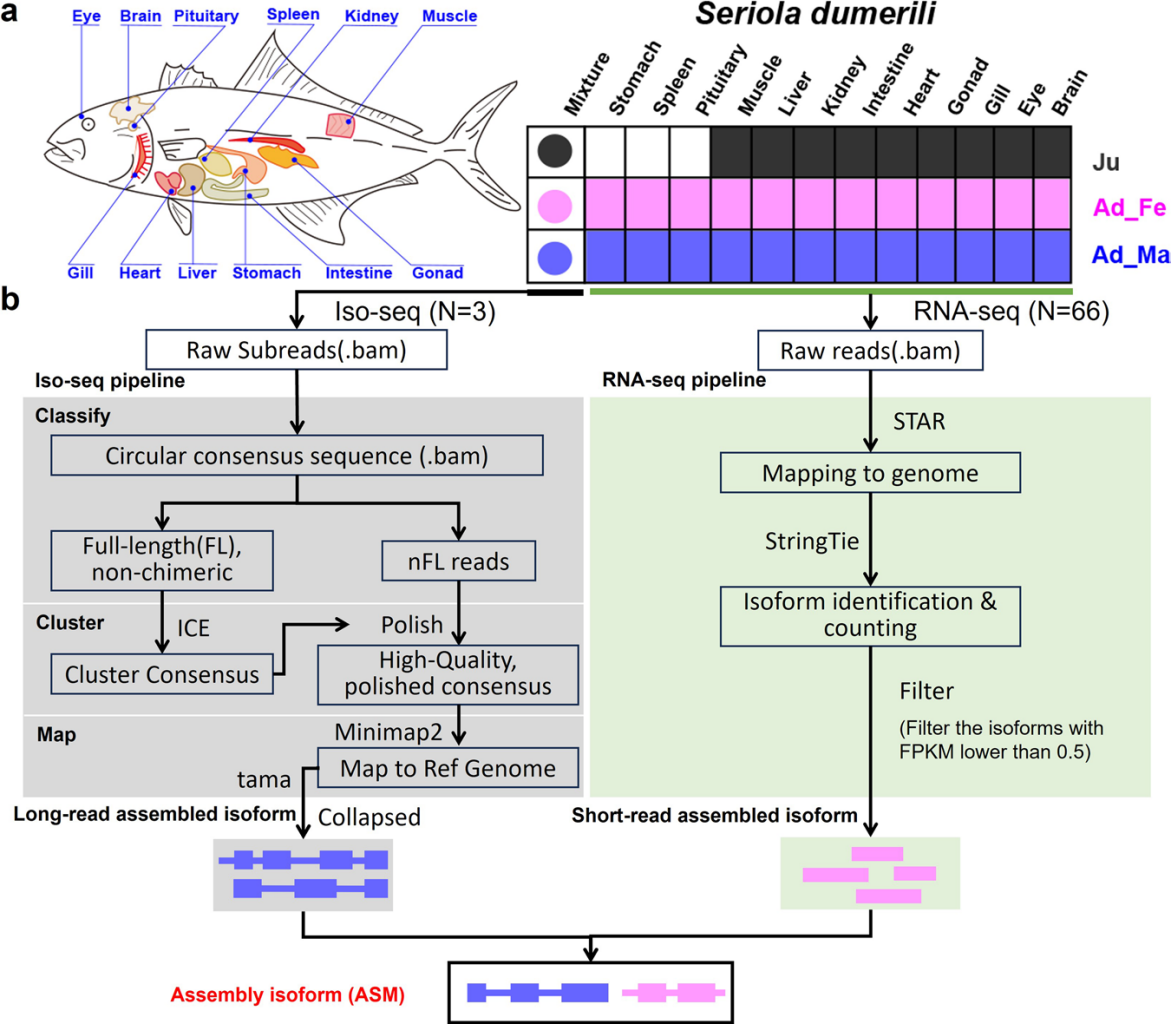
Schematic overview of the generation of the comprehensive gene annotation. (**a**) Samples were harvested from 12 tissues eye, brain, pituitary, heart, muscle, spleen, gill, liver, intestine, stomach, gonad, and kidney at both the juvenile and adult stages for both sexes to conduct next-generation RNA sequencing (RNA-seq) and full-length isoform sequencing (Iso-seq). Ju, Ad_Fe, and Ad_Ma represent juvenile *S. dumerili*, adult female *S. dumerili*, and adult male *S. dumerili*, respectively. (**b**) The analysis pipeline for Iso-seq, RNA-seq, and generating assembly isoforms.

The greater amberjack individuals were obtained from Hainan Yonghe Biotechnology Co., Ltd. (Hainan, China) and Fujian Dongshan Fuminyang Aquatic Development Co., Ltd. (Guangdong, China). Twelve tissues (eye, brain, pituitary, heart, muscle, spleen, gill, liver, intestine, stomach, gonad, and kidney) were harvested for RNA sequencing. The sex of juveniles cannot be determined, so we only differentiated the sex of adult fish. Additionally, harvesting pituitary, spleen, and stomach tissues from juvenile fish is challenging, so we did not include these tissues in our juvenile sample set. For each tissue, a paired-end RNA-sequencing library was constructed with an insert size of 300 bp, and then the library was sequenced on the MGI DNBSEQ platform to generate paired-end (PE) reads of 2 × 150 bp. Each tissue was represented by two biological replicates. Three mixtures (juveniles, adult females, and adult males) were also used to construct an Iso-seq library with fragment sizes of 5–10 K, which was subsequently sequenced on the PacBio RSII platform (Fig. 1). We used the greater amberjack FASTA genome from NCBI (ID: GCF_002260705.1)^13^, the GTF annotation file from NCBI (ID: GCF_002260705.1)^13^, and the GTF annotation file from Ensembl (ID: Sdu_1.0.103)^14^.

### Short-read assembly using RNA-seq

The adapter sequences were removed by BGI prior to delivery. The analysis of the phred quality score distribution of the RNA-seq data was conducted using the numpy and pandas packages in Python. To improve the precision and sensitivity of splice junction discovery, paired-end DNBSEQ RNA-seq reads were first aligned to the greater amberjack genome (Sdu_1.0) by using STAR (v2.7.5b)^15^ software. This alignment was performed in end-to-end mode, employing the following parameters “–runMode alignReads–outSAMtype BAM SortedByCoordinate–limitBAMsortRAM 10000000000–readFilesCommand zcat –outFileNamePrefix –genomeDir –genomeLoad–readFilesIn”. Then, the reads were aligned to the genome with splice junctions from the 12 tissues. The statistical mapping results that were obtained using STAR software were analyzed using the numpy and pandas packages in Python. Only the uniquely mapped alignments were considered for further analysis. The potential PCR amplification bias that was introduced during library construction was approximately removed by using the MarkDuplicates command of the Picard Toolkit (v2.23.3, http://broadinstitute.github.io/picard/). The Fragments Per Kilobase of exon model per Million (FPKM) values and counts for different genomic features were calculated using StringTie (v2.1.4)^16^ with the following parameters: “–rf -e -A gene_abund.tab -C cov_refs.gtf -G input.gtf -o transcripts.gtf”.

### Long-read assembly using PacBio full-length Iso-seq

We utilized several programs from PacBio SMRT Analysis and followed the SMRT Tools Reference Guide in this processing pipeline. For each transcriptome library, circular consensus sequences (CCSs) were constructed from the raw subreads by using ccs (v6.0.0) with the following parameters “–skip-polish–min-passes 4–min-length 200–min-rq 0.99”. The primer sequences in the long CCS reads were removed by using lima (v2.0.0) with the parameter “–isoseq”. The full-length, nonchimeric (FLNC) cDNA reads were identified by the refine subcommand of isoseq. 3 (v3.4.0) with the following parameters: “–require-polya–min-polya-length 20”. Then, we used the cluster and polish subcommand to cluster FLNC reads and generated polished transcripts. Finally, the polished transcripts were mapped to the greater amberjack genome (Sdu_1.0) by using minimap2 (v2.17-r941)^17^ with the parameter “-x splice”. During SMART processing, we used the numpy and pandas packages in Python to analyze the CCS reads and generate statistical results, as well as to determine the length distribution of high-quality unique transcript sequences.

### Construction of gene annotation and validation of the polyA site

The ultimate gene annotation was generated from the integration of the isoforms identified by RNA-seq and Iso-seq. Since long-read assembled isoforms reveal the original structures of transcripts, all long-read assembled isoforms were retained. The short-read assembled isoforms were filtered out according to the following criteria: a) overlap with long-read assembled isoforms in the same orientation and b) low expression level (FPKM lower than 0.5 in all samples). The remaining short-read assembled isoforms were merged with long-read assembled isoforms to produce the comprehensive gene annotation. In detail, for RNA-seq, we used StringTie (v2.1.4)^16^ with the parameter “–rf” to construct isoforms *de novo* from short-read alignments and merged the assemblies. For Iso-seq, we collapsed the full-length alignments to construct isoforms using a script from TAMA with the parameter “tama_collapse.py -s output.sam -f input.fa -x no_cap -p params.prefix” and merged the assemblies using the tama_merge.py script. The gene character analysis was conducted using the numpy and pandas packages in Python.

The transcripts were identified as internal primer transcripts and removed if the 20nt sequence downstream of the polyA site contained more than 15As. The retained isoforms were termed long-read assembled isoforms. The final assembly model (ASM) was predicted using the long-read assembled isoforms and supplemented with short-read assembled isoforms that did not overlap with long-read assembled isoforms on the same strand. Finally, all the transcriptome isoforms were compared to the NCBI annotation using SQANTI3 (v3.6.1)^18^ to identify genes and fusion genes. For the NCBI annotation, the version of the assembly and gene model annotation that were used was Sdu_1.0. and GCF_002260705.1_Sdu_1.0, respectively.

### Functional annotation of protein-coding genes

The ORFs of the isoforms were predicted using ORFfinder (v0.4.3), and the translated amino acid sequences were aligned to the NCBI NR databases using the Diamond (v0.9.14.115)^19^ program with an e-value < 1e-5. A hit with more than 90% identical matches and the lowest e-value for a certain ORF was considered to be a homologous protein. For the isoforms with more than one predicted ORF, we selected the ORF with the lowest e-value as the final ORF. The GO terms and KEGG pathways were assigned by eggNOG-mapper (v2)^20^ with the following parameter: “emapper.py -m diamond -i input.fa–output params.prefix -d euk–usem”.

### Identification of tissue-specific genes in the greater amberjack

Tissue-specific genes are defined as those that exhibit higher expression exclusively within a particular tissue. FPKM is calculated with the following formula: $FPKM=\frac{count\times {10}^{9}}{Librarysize\times Length}$. Initially, genes with an FPKM less than 1 in all the samples were filtered out. Subsequently, the z score for each gene across all the samples was computed. Genes with a z score greater than 1 in a specific tissue and a z score less than 0 in other tissues were classified as tissue-specific genes. The following formula was used for calculating the z-score^21^: $z=\frac{{\rm{x}}-{\rm{\mu }}}{{\rm{s}}}$, where *x* is the gene expression, *μ* is the mean of all gene expression across the 12 tissues, and *s* is the standard deviation. Short reads from RNA-seq were aligned to the ASM, and gene expression levels were calculated using StringTie with the following parameters: “–rf -e -A output.out/gene_abund.tab -C output.out/cov_refs.gtf -G input.gtf -o”. The correlation calculation was performed using the clustermap function from the seaborn package in Python (https://seaborn.pydata.org/generated/seaborn.clustermap.html), and the clustering algorithm was hierarchical/agglomerative clustering.

## Data Records

All the RNA-seq and full-length Iso-seq raw reads and relevant information for the greater amberjack were deposited in the Sequence Read Archive (SRA) of the National Center for Biotechnology Information under BioProject accession number PRJNA1035924^22^. The transcriptome GTFs, FASTA and SQANTI reports for short-read assembled isoforms, long-read assembled isoforms, and our assembled isoforms can be accessed through the Zenodo under 10.5281/zenodo.11207647^23^.

**Fig. 2:**
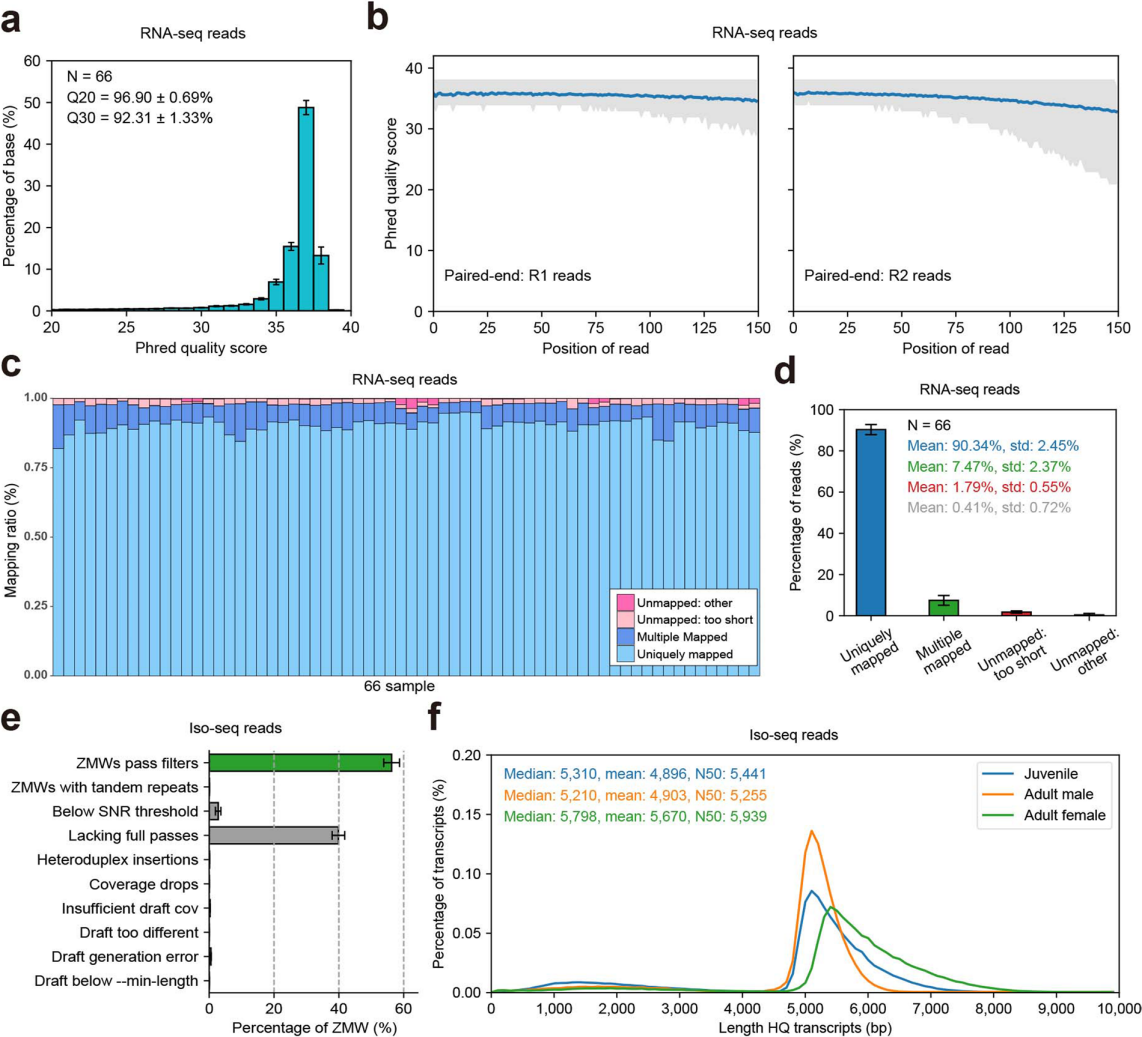
Summary of sequencing and genome alignment data. (**a**) The distribution of the mean quality of the RNA-seq reads. (**b**) The base quality of the paired-end R1 and R2; 10,000 reads were randomly extracted from each sample. (**c,d**) Summary of genome alignment for RNA-seq. Unique: reads successfully mapped to one genomic location; Multiple: reads successfully mapped to multiple genomic locations; Short: reads that failed to be mapped because the read was too short; Other: reads that failed to be mapped for other reasons. (**e**) Sequencing data of high-quality reads for Iso-seq after filtering out adapters, chimeric sequences, and low-quality sequences. (**f**) The length distribution of the high-quality unique transcript sequences for Iso-seq. Blue, orange, and green represent juvenile, adult male, and adult female *S. dumerili*, respectively.

For RNA-seq, we performed strand-specific RNA-seq (paired-end, 150 bp), generating a total of 3,425,467,779 (3.42 Gb) clean read pairs, averaging 51,901,027 (51.9 ± 6.3 M) pairs per cDNA library (Table 1). Among these reads, 92.31% had a mean base quality higher than or equal to the Q30 (Fig. 2a). Furthermore, we randomly extracted 10,000 reads from each sample and calculated the base quality of the paired-end R1 and R2, both of which had an average score of 37 (Fig. 2b), indicating a very high prediction accuracy for the base call. On average, 90.34% of the short reads were aligned to a unique location of the genome, and an additional 7.47% of the short reads were aligned to multiple locations, indicating that the collected RNA was almost free of contamination (Fig. 2c,d and Table 1). After genome alignment and removal of PCR duplicates, we obtained 1,658,051,483 (1.66 Gb) read pairs, with an average of 25,121,992 pairs of reads per cDNA library. In the short-read approach, we assembled 91,901 isoforms (short-read assembled isoforms) of 51,687 genes, averaging 3 isoforms per gene and 13 exons per isoform. Among these genes, 17,491 (33.84%) contained multiple isoforms (>2 isoforms). Among these isoforms, 92,811 (87.34%) contained multiple exons (Figure S1a).

**Table 1:** Summary of sequencing and genome alignment results for RNA-seq.

Tissue	Juvenile				Adult male				Adult female			
	Replicate 1		Replicate 2		Replicate 1		Replicate 2		Replicate 1		Replicate 2	
	Read Pairs	Mapped	Read Pairs	Mapped	Read Pairs	Mapped	Read Pairs	Mapped	Read Pairs	Mapped	Read Pairs	Mapped
Brain	50,708,476	88.17%	50,701,782	90.52%	51,519,111	92.13%	40,865,882	87.40%	41,090,797	81.93%	41,033,467	86.83%
Eye	50,308,846	90.49%	50,341,739	91.73%	51,215,267	90.48%	39,023,075	88.83%	40,383,580	87.46%	40,451,467	89.14%
Gill	50,667,828	91.88%	51,135,294	91.81%	48,890,128	90.75%	50,258,336	92.13%	61,171,646	90.68%	60,827,232	91.84%
Gonad	50,442,747	92.56%	50,979,610	93.29%	51,277,725	93.27%	40,026,759	91.41%	50,176,177	91.39%	50,724,918	91.04%
Heart	46,854,864	84.96%	50,685,703	84.64%	50,867,074	88.91%	50,598,194	88.60%	61,591,606	86.90%	60,467,968	84.50%
Intestines	50,495,881	91.53%	46,032,123	91.47%	46,856,328	92.26%	50,718,308	90.14%	61,213,278	91.50%	61,115,549	91.30%
Kidney	49,021,431	89.38%	47,740,745	89.94%	50,597,947	89.50%	50,705,742	88.69%	60,911,597	89.93%	61,887,636	88.33%
Liver	50,159,451	91.37%	50,479,484	91.08%	46,134,672	90.87%	50,921,036	91.16%	61,238,228	91.57%	61,403,224	91.99%
Muscle	50,121,664	88.35%	46,964,652	87.72%	48,177,451	91.50%	51,189,782	91.18%	60,992,914	90.78%	61,673,903	88.81%
Pituitary	—	—	—	—	51,455,009	95.06%	50,598,604	94.80%	61,106,496	94.61%	60,652,131	94.73%
Spleen	—	—	—	—	51,240,288	91.52%	51,051,550	91.39%	61,420,845	89.09%	62,226,005	90.05%
Stomach	—	—	—	—	49,064,436	90.53%	50,482,665	91.49%	53,611,780	91.16%	60,517,646	91.82%

Reads: clean read number. Mapped: percentage of uniquely mapped to genome.

For PacBio full-length Iso-seq, a total of 17,409,419 (17.41 M) polymer reads were yielded (with an average of 15 subreads per polymer read) (Table 2). Of these, 53.67%, 56.89%, and 58.42% of the polymer reads from juvenile, adult male and adult female *S. dumerili*, respectively, were successfully combined into circular consensus sequences (CCS). After SMRT-link processing (see Method), approximately 60% of the reads passed through a zero-mode waveguide filtering (ZMWs, Fig. 2e), and 163,218 (163.3 K), 149,716 (149.7 K), and 189,169 (189.2 K) high-quality (HQ) unique transcript sequences were obtained, with an N50 of 5,441, 5,255, and 5,939, respectively, for each library (Fig. 2f and Table 2). More than 99.9% of HQ transcript sequences were successfully aligned to the greater amberjack genome. In the long-read approach, we assembled 130,734 isoforms (long-read assembled isoforms) of 17,457 genes, averaging nearly 8 isoforms per gene and 17 exons per isoform. Among these genes, 11,608 (66.49%) contained multiple isoforms. Among these multiple isoforms, 119,012 (95.88%) contained multiple exons (Figure S1a).

**Table 2:** Summary of sequencing and processing for Iso-seq.

Library	Bases (Gb)	Polymerreads	Subreads/polymerread	Circular Consensus Sequencing			FLNC(PolyA+)	Cluster and Polished	
				CCS	CCS (%)	N50		HQ transcripts	N50
Juvenile	384.99	6,433,289	14.7	3,452,684	53.67%	5,541	2,339,901	163,218	5,441
Adult male	364.85	5,215,938	15.75	2,967,394	56.89%	5,369	1,887,924	149,716	5,255
Adult female	420.74	5,760,192	15.34	3,364,862	58.42%	6,031	2,381,123	189,169	5,939

## Technical Validation

### Quality control of comprehensive gene annotation

**Fig. 3:**
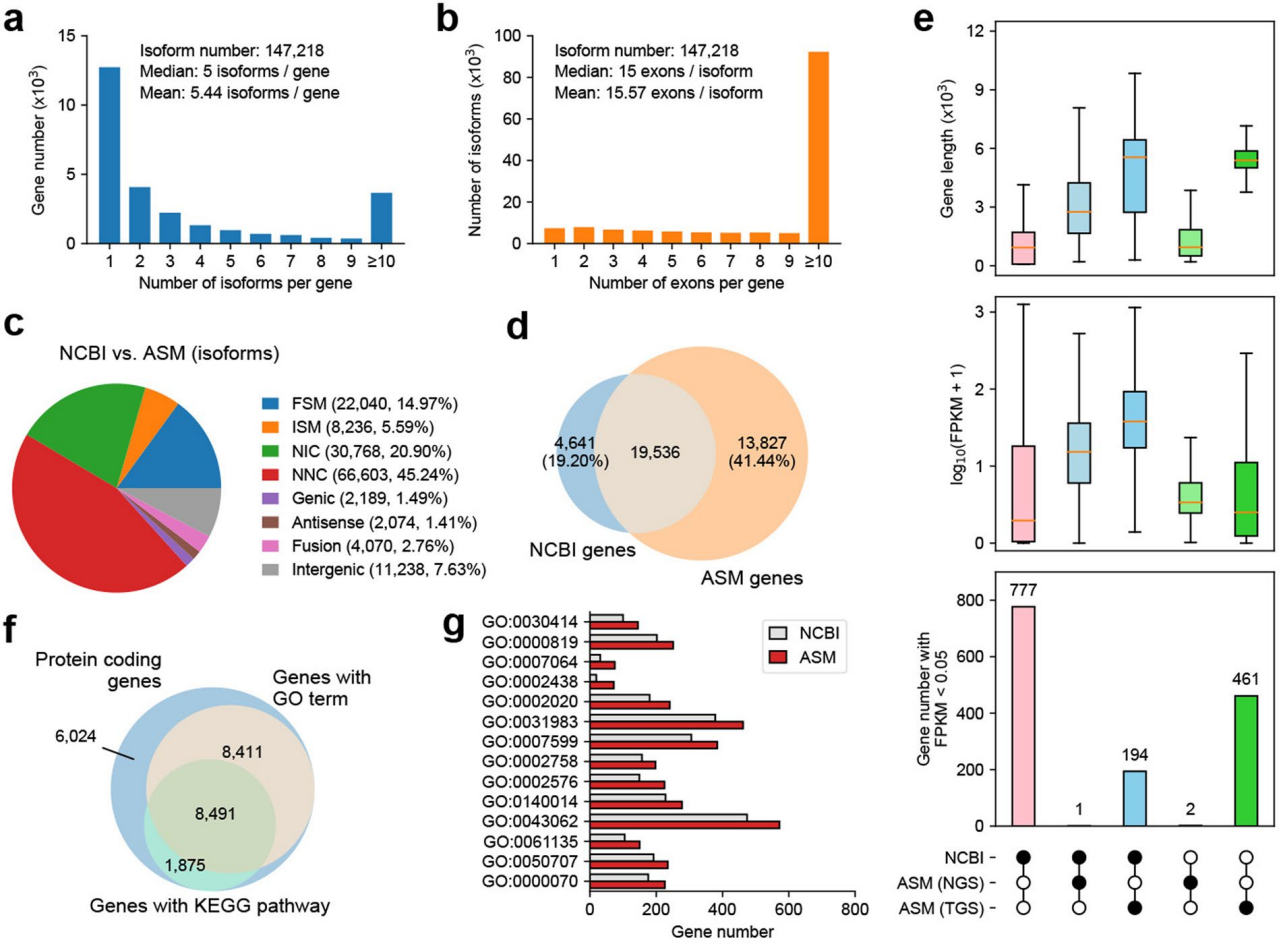
Comprehensive gene annotations in our assembled transcriptome. (**a**) Number of isoforms per gene in our assembled transcriptome. (**b**) Number of exons per isoform in our assembled transcriptome. (**c**) The proportions of SQANTI structural categories in our assembled isoforms compared to those in the NCBI annotation. FSM: matches all splice junction (SJs) perfectly; ISM: matches the reference SJs partially; NIC: novel isoform with a new combination of known splice sites; NNC: novel isoforms with at least one new splice site; Genic: within an intron or overlaps introns and exons; Antisense: in the reverse strand relative to the reference isoform; Fusion: overlap with isoforms from more than 1 gene; Intergenic: do not overlap with any isoform. (**d**) Overlapping genes between NCBI annotation and our comprehensive annotation. (**e**) The distribution of gene length, gene expression based on the FPKM value, and the number of genes with low expression in different gene sets. ASM (NGS) and ASM (TGS) represent genes within our assembled transcriptome that were assembled from short reads and long reads, respectively. (**f**) The genes that were successfully assigned to GO terms and KEGG pathways. A total of 16,902 genes were successfully assigned to GO terms, and 10,366 genes were assigned to KEGG pathways. Of these, 8,411 genes were only associated with GO terms, 1,875 genes were exclusively associated with KEGG pathways, and 8,491 genes were associated with both GO term and KEGG pathway. (**g**) Comparison of the number of genes assigned GO terms between the NCBI reference genome and our assembly (ASM) annotation. ASM annotation was identified for GO terms, where the number of enriched genes is more than 1.2 times that of NCBI database, with a difference greater than 40.

Comprehensive gene annotation was achieved by integrating RNA-seq and Iso-seq data. The assembly statistics are shown in Fig. 3 and Table 3. Compared with the NCBI and Ensembl isoforms, the novel annotations identified more than 3 times more isoforms, and the isoforms were longer (Figure S1a). Furthermore, long-read assembled isoforms exhibited a higher number of isoforms per gene, demonstrating the robust capability of full-length isoform sequencing for detecting isoforms (Figure S1a). Short-read assembled isoforms tended to have fewer exons (1–5 exons per isoform), whereas long-read assembled isoforms often contained more exons (over 10 exons per isoform) (Figure S1b).

**Table 3:** Summary of gene annotations from different sources.

Build	Isoform numbers	Gene numbers	Isoform per gene	Gene with multi isoforms	Exon per isoform	Isoform with multi exons	Isoform length		
							Mean	Median	N50
NCBI	34,971	23,878	1.46	6,121 (25.63%)	11.57	33,051 (94.51%)	2748.62	2,333	3,537
ENSEMBL	33,717	23,808	1.42	6,504 (27.32%)	10.53	32,323 (95.87%)	2553.64	2,004	3,700
NGS	91,901	30,549	3.01	14,983 (49.05%)	13.11	88,629 (96.43%)	4542.42	3,945	6,415
TGS	130,734	17,527	7.46	11,597 (66.17%)	16.86	126,052 (96.42%)	5300.74	5,458	5,588
ASM	147,218	27,087	5.44	14,353 (52.99%)	15.57	139,832 (94.98%)	4971.84	5,381	5,563

Our assembly results (ASM-based isoforms) identified a total of 147,218 isoforms from 45,167 genes across all 12 tissues, with an average of 5.44 isoforms per gene (Fig. 3a) and 16 exons per isoform (Fig. 3b). In the comparison between our assembly and the NCBI annotation, 22,040 (14.97%) isoforms corresponded to full splice matches (FSMs) in the NCBI annotation, and 66,603 (45.24%) isoforms contained novel splice sites (Novel Not in Catalog, NNC) (Fig. 3c). This proportion of NNC sites in the ASM-based isoforms was lower than that in both the short-read assembled isoforms and long-read assembled isoforms compared with that in the NCBI and Ensembl databases, demonstrating more transcripts matching known reference transcript in the ASM-based transcriptome; this also serves as one of the indicators of a more complete assembly of our transcriptome (Figure S1c). A significant increase of NIC (Novel in Catalog) isoforms in the long-read transcriptome compared to short-read transcriptome (Figure S1c), demonstrating an advantage of the long-read transcriptome. Among these genes, 13,827 (41.44%) were novel genes that were not annotated in the NCBI database (Fig. 3d). According to gene character analysis, genes that were annotated only in our assembly from short-read assembled isoforms or in the NCBI database were typically shorter and expressed at lower levels than those that were annotated from both sources. In contrast, genes that were identified in both our assembly and the NCBI database tended to be characterized by longer lengths and higher expression (Fig. 3e). To determine the functions of these genes, we extracted the predicted ORF sequences and searched the NR, eggNOG, Pfam, Swiss-Prot, KOG, GO, KEGG, and COG databases. Overall, 24,801 (74.33%) genes were annotated as protein-coding genes. Among the protein- coding genes, 16,902 (68.15%) genes were successfully assigned to GO terms, and 10,366 (41.80%) genes were assigned to KEGG pathways (Fig. 3f). Within specific GO terms, the number of genes that were identified in the ASM annotation surpassed that in NCBI, as indicated by values exceeding the red line (Fig. 3g).

### Quality control of polyA site location

**Fig. 4:**
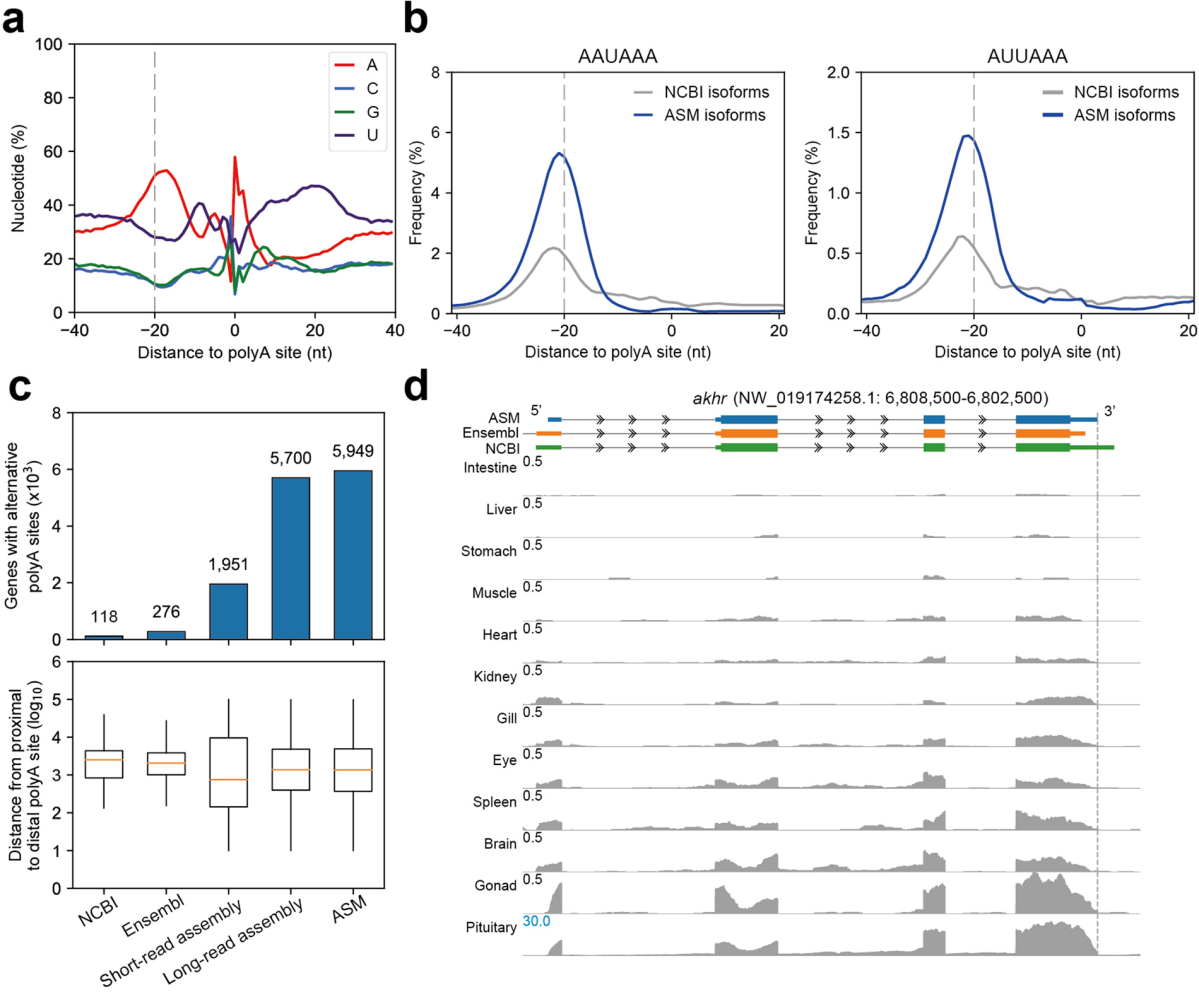
Annotation of the precise location of polyA sites. (**a**) Nucleotide content around transcription stop sites according to comprehensive gene annotation. (**b**) Density of polyA processing signals around transcription stop sites according to comprehensive gene annotation. This frequency indicates the proportion of transcription stop sites where these motifs (AAUAAA and AUUAAA) are located at a specific upstream position, using the first base of the motif as the reference point for the statistics. (**c**) The number of genes that contain canonical alternative polyadenine (top) and the distribution of the distance between the proximal and corresponding distal polyA sites at different annotations (bottom). (**d**) RNA-seq agreement with polyA sites defined by Iso-Seq. Blue, orange, and green represent the *akhr* transcripts from our assembled transcriptome, the Ensembl database, and the NCBI database, respectively. The gray track represents the expression coverage of the *akhr* transcript across the 12 tissues based on the IGV. The gray dashed line represents the position of the 3′UTR of the *akhr* transcript in our assembled transcriptome, which precisely aligns with the end of the last exon of the *akhr* mRNA in the 12 tissue samples. The transcript ID of the *akhr* gene from the Ensembl is ENSSDUT00000005875, and the gene_id is ENSSDUG00000004223. The transcript ID of the *akhr* gene from the NCBI is XM_022740281.1, and the gene_id is LOC111218147. The transcript ID of the *akhr* gene from the ASM is G610.2, and the gene_id is G610.

To evaluate the 3′ end of our assembled transcriptome model, we analyzed the nucleotides content surrounding the putative polyA site. The results revealed a tendency of high contents of adenine (A) in the 20 base pairs (bp) upstream of the polyA site, while the 20 base pairs downstream of the polyA site were rich in uracil (U). In the comparison isoforms from different sources, long-read assembled isoforms showed the most significant tendency, while those from Ensembl displayed an almost random distribution of nucleotides (Figure S2a–d). This regularity observed in the Ensembl is due to over half of the isoforms not containing a 3′UTR and confirmed the completeness of the isoforms assembled in ASM transcriptome. Furthermore, we calculated the density of conserved 3′ end processing signals around the polyA sites and found enrichment of AAUAAA and AUUAAA in the 20 bp upstream of the polyA site compared with the NCBI annotations (Fig. 4b).

The identification of genes with a certain distance between the proximal and distal polyA sites is an indicator of the completeness of our assembled transcriptome. Transcripts with varying 3′ UTR lengths are thought to play a role in regulating gene expression at the posttranscriptional level by influencing RNA stability, translation efficiency, or cellular localization. We determined the distance between the proximal polyA site and the distal polyA site for genes with multiple isoforms. The number of genes with Alternative Polyadenylation (APA) (with a distance >100 bp) from the ASM-based isoforms was counted and compared with that from the long-read assembled, short-read assembled, NCBI, and Ensembl results. ASM-based isoforms included 5,949 genes with APA, which was far more than others (Fig. 4c). However, the distribution of distance among the genes with APA did not significantly differ among the assemblies (Fig. 4d). To verify the precision of the polyA site annotation, we showed the tracks of *akhr* gene across the 12 tissues (Fig. 4d). The polyA sites of *akhr* were found to vary among the data from NCBI, Ensembl, and our assembly. Our assembly provided greater precision in locating polyA sites than existing gene models and revealed the complexity of the transcriptome at alternative polyadenylation level.

### Correlation analysis between samples based on gene expression profiling

**Fig. 5:**
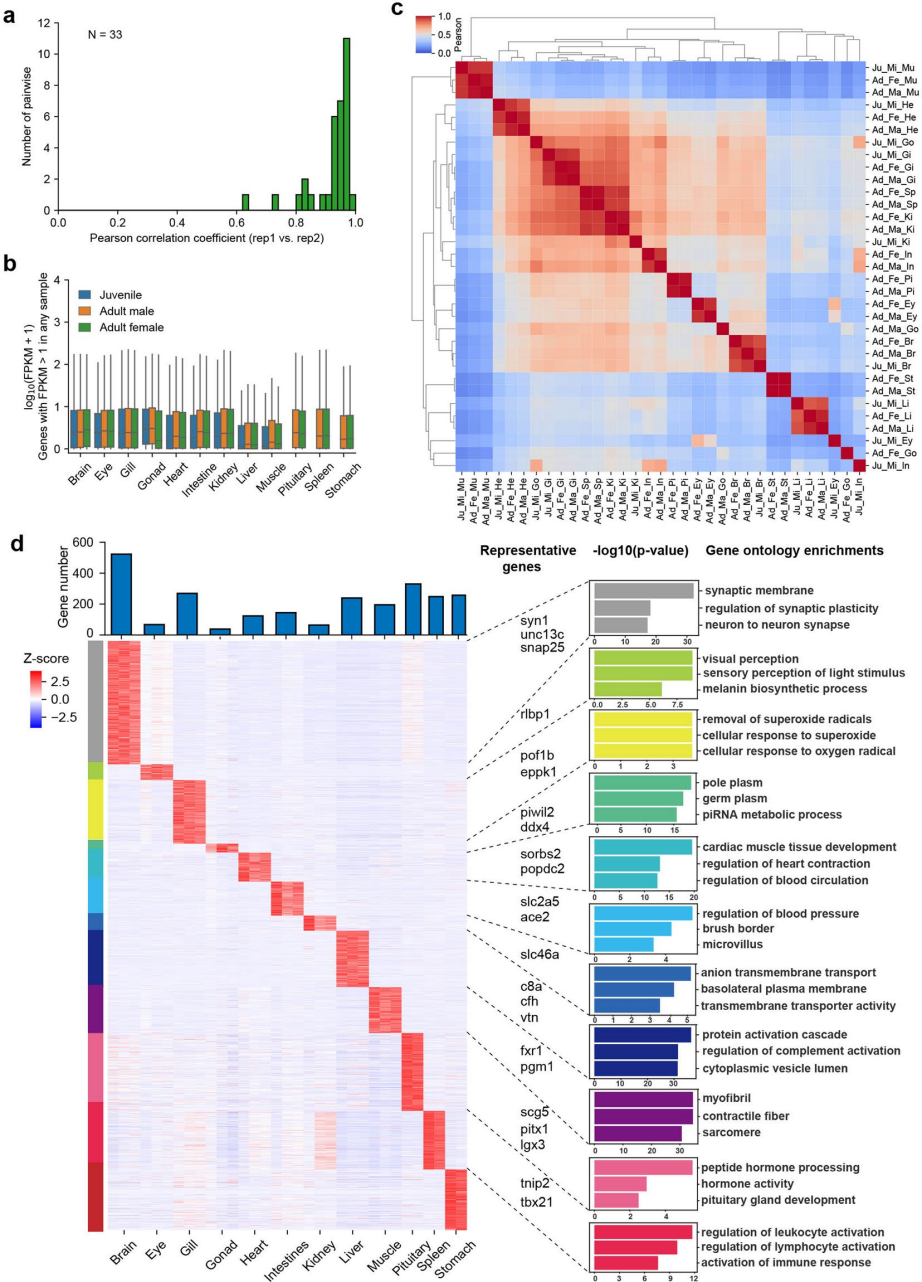
Gene expression profile in 12 tissues. (**a**) Correlation analysis between two biological replicates within 33 groups. (**b**) The distribution of FPKM across all samples. (**c**) Correlation analysis among all 66 samples, including both intragroup and intergroup samples, based on FPKM values. (**d**) Relative expression and biological functions of tissue-specific genes. The number of tissue-specific genes is shown at the top of the heatmap; representative genes and the top 3 GO terms for tissues-specific genes are shown at the right of the heatmap. Note that the stomach tissue-specific genes did not enrich for any GO terms.

Based on the improved gene annotation, we attempted to construct a gene expression profile for the greater amberjack. Short-read data were used to quantify gene expression across the 12 tissues at two developmental stages (juvenile and adult) and from different sexes (male and female). Each sample had two biological replicates. Furthermore, the fragments per kilobase per million mapped reads (FPKM) for our assembled genes were computed using the short-read data, and their similarity and specificity were investigated. The results demonstrated that the correlation coefficients of two biological replicates within a group were mostly greater than 0.8 (Fig. 5a). The FPKM distribution across the 12 tissues showed that the FPKM values from the liver and muscle were slightly lower than those from the other 10 tissues (Fig. 5b). The correlation analysis results of all the samples indicated that, except for the intestines and eyes of the juveniles, the correlation coefficients within the tissues of juveniles, adult females and adult males all reached 0.9, indicating that the samples clustered together well (Fig. 5c).

We identified the genes that were uniquely highly expressed in each tissue and generated a z score normalized expression heatmap clustered by genes. In the present study, tissue-specific genes clustered together, showing similar expression patterns and significantly higher expression levels than those that were expressed in multiple tissues (Fig. 5a). Of all the tissues, the brain had the most tissue-specific genes (524), followed by the pituitary gland (349), spleen (287), gill (282), stomach (270), and liver (245). On the other hand, the gonad (39) and eye (74) had the fewest tissue-specific genes (Fig. 5a). GO term enrichment analysis of these genes was performed based on our functional annotation. The terms that were enriched in certain tissues were consistent with the functions of the tissues; for example, the brain-specific genes enriched neuron-related terms, such as “synaptic membrane”, “regulation of synaptic plasticity” and “neuron to neuron synapse”; the pituitary gland-specific genes enriched endocrine-related terms, such as “peptide hormone processing”, “hormone activity” and “pituitary gland development”; and the gonad-specific genes enriched the reproduction-related terms, such as “pole plasm”, “germ plasm”, and “piRNA metabolic process” (Fig. 5a). The details of the enriched GO terms for each tissue are summarized in Table 3. These findings prove that our results are credible and can be used to guide further research.

## Usage Notes

The ASM dataset was constructed with a comprehensive gene annotation that not only had greater detail and accuracy than those from NCBI and Ensembl, but also featured more precise polyA site locations, as well as systematically profiled gene expression patterns across the 12 tissues in the greater amberjack. The uploaded binary alignment (BAM) files contain reads from the short-read assembled, long-read assembled, NCBI, Ensembl, and ASM that were already mapped to reference genome of the greater amberjack. These aligned files can be further analyzed using various bioinformatics program packages, such as STAR, stringtie, TACO, CCS, lima, isoseq3, pbmm2, minimap2, tama, bedtools, ORFfinder, diamond, eggNOG, sqanti3, and samtools, or visualized using, e.g., IGV. The uploaded Illumina and PacBio files were not trimmed and contain terminal poly(A) sequences as well as 5′ and 3′ adapter sequences, which can be used to determine the orientations of the reads.

### Supplementary information

Supplementary Information

## Data Availability

All the software that was used in this study is publicly available, and the parameters that were used are clearly described in the Methods sections. If no detailed parameters were mentioned for a particular software, the default parameters were used as suggested by the developer. The code for the analysis pipelines was deposited at GitHub: https://github.com/Ckenen/integrated-transcriptome-of-seriola-dumerili.
