# Supplementary material for: Integrating Iso-seq and RNA-seq data for the reannotation of the greater amberjack genome
Source: Sci Data. 2024 Jun 22;11:675. doi: 10.1038/s41597-024-03495-7 (PMC11193819; doi:10.1038/s41597-024-03495-7)
Supplement: Supplementary file 1 — Supplementary Information [file 41597_2024_3495_MOESM1_ESM.pdf]

Contents

Supplementary Figure 1 1

Supplementary Figure 2 2

Supplementary Figure 1: The metrics of gene annotations.

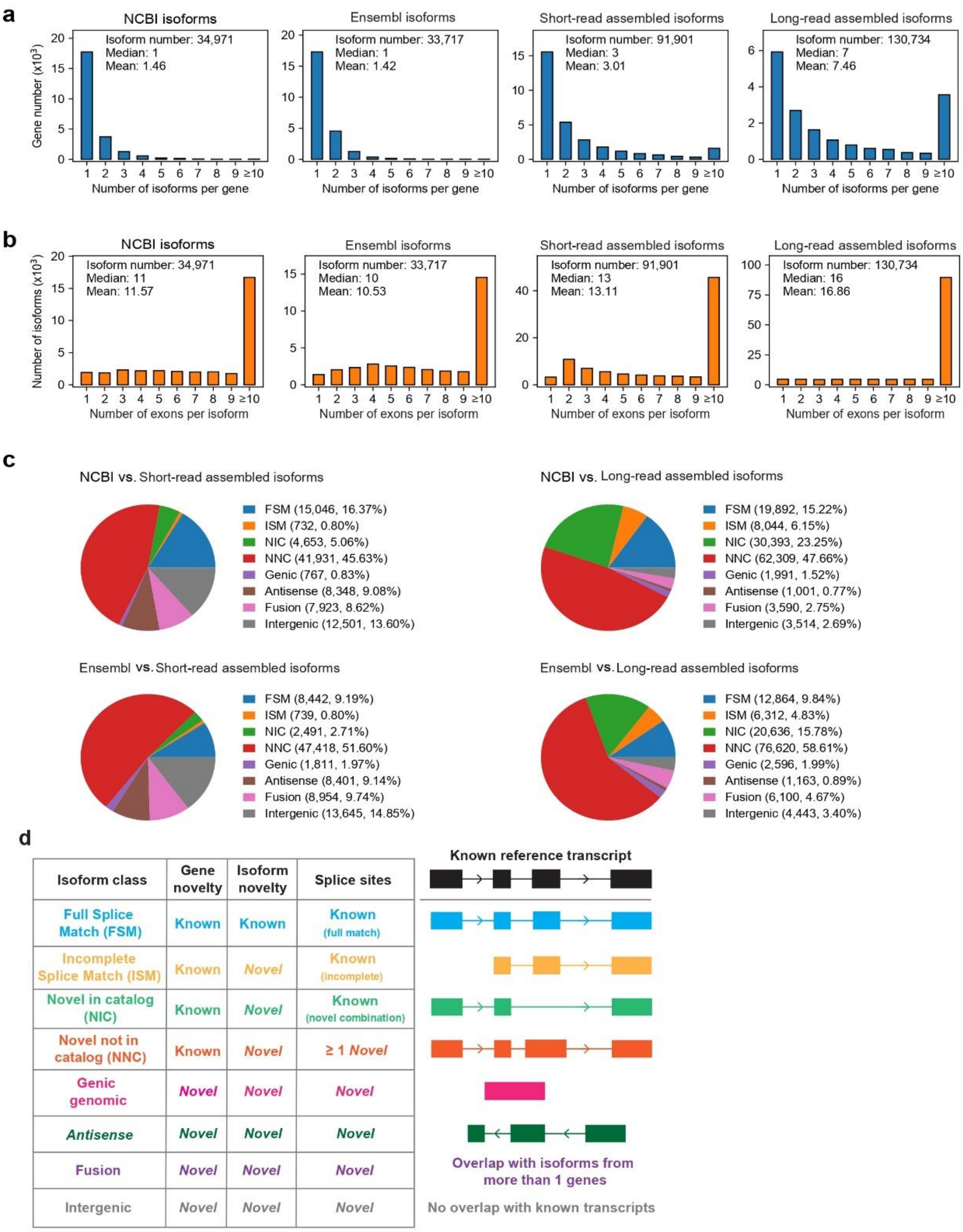

a: Comprehensive analysis of the number of isoforms per gene for short-read assembled, long-read assembled, NCBI, and Ensembl isoforms.

b: Comprehensive analysis of the number of exons per isoform for short-read assembled, long-read assembled, NCBI, and Ensembl isoforms.

c: Proportion of SQANTI structural categories for short-read assembled isoforms and long-read assembled isoforms compared with the NCBI and Ensembl annotations. FSM: matches all splice junction (SJs) perfectly; ISM: matches the reference SJs partially; NIC: novel isoform with a new combination of known splice sites; NNC: novel isoforms with at least a new splicing site; Genic: within an intron or overlaps introns and exons; Antisense: in the reverse strand to the reference isoform; Fusion: overlap with isoforms from more than 1 gene; Intergenic: do not overlap with any isoform.

d: Schematic diagrams of different classes of transcripts generated from SQANTI3.

Supplementary Figure 2: The metrics of polyA site annotations.

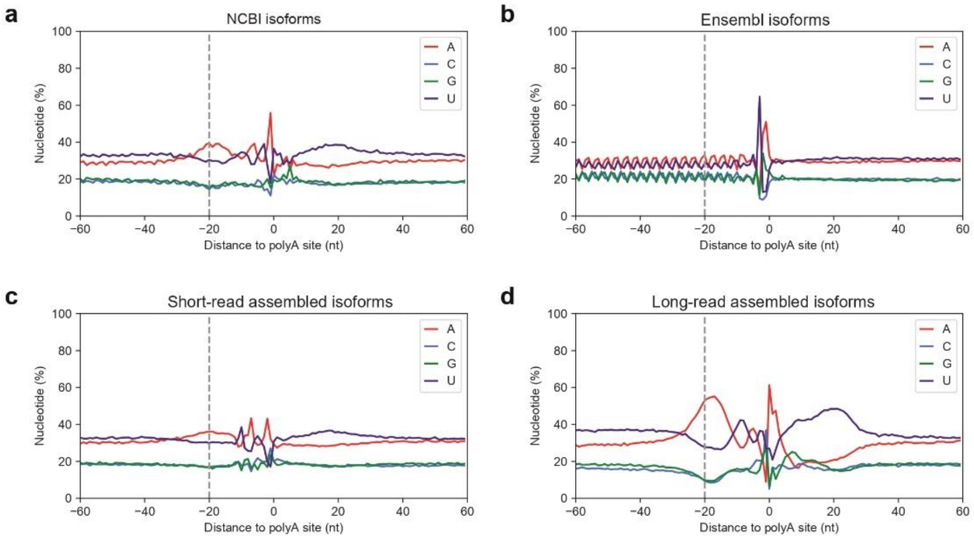

a-d: Nucleotide content around transcription stop sites in the NCBI, Ensembl, short-read assembled, and long-read assembled isoforms.
